# Neural Plasticity of Mild Tinnitus: An fMRI Investigation Comparing Those Recently Diagnosed with Tinnitus to Those That Had Tinnitus for a Long Period of Time

**DOI:** 10.1155/2015/161478

**Published:** 2015-07-13

**Authors:** Jake R. Carpenter-Thompson, Sara A. Schmidt, Fatima T. Husain

**Affiliations:** ^1^Neuroscience Program, University of Illinois at Urbana-Champaign, Champaign, IL 61801, USA; ^2^Medical Scholars Program, University of Illinois at Urbana-Champaign, Champaign, IL 61801, USA; ^3^Beckman Institute, University of Illinois at Urbana-Champaign, Champaign, IL 61801, USA; ^4^Department of Speech and Hearing Science, University of Illinois at Urbana-Champaign, Champaign, IL 61820, USA

## Abstract

*Objectives*. The aim of the study was to compare differences in neural correlates of tinnitus in adults with recent onset and others who had the disorder for longer than a year.* Design*. A total of 25 individuals with tinnitus were divided into groups based on the amount of time for which they had experienced tinnitus: <1 year (RTIN) or >1 year (LTIN). Subjects underwent an fMRI scan while listening to affective sounds from the International Affective Digital Sounds database. Resting state functional connectivity data were also collected.* Results*. The RTIN group recruited the posterior cingulate and insula to a greater extent than the LTIN group when processing affective sounds. In addition, we found that the LTIN group engaged more frontal regions when listening to the stimuli compared to the RTIN group. Lastly, we found increased correlations between the default mode network and the precuneus in RTIN patients compared to LTIN at rest.* Conclusion*. Our results suggest that the posterior cingulate and insula may be associated with an early emotional reaction to tinnitus in both task and resting states. Over time, tinnitus patients may recruit more frontal regions to better control their emotional response and exhibit altered connectivity in the default mode network.

## 1. Introduction

Tinnitus, the perception of noise without an external sound stimulus, is estimated to affect more than 50 million adults in the United States [1]. Tinnitus is associated with impaired sleep habits, difficulty concentrating, and, in severe cases, suicide [2–4]. Given the prevalence of tinnitus and its detrimental effect on sufferers, it is important to investigate the underlying neural correlates involved in tinnitus. Past studies have linked increased activation in the limbic system to tinnitus [5–9]. Although a number of studies have investigated the neural correlates associated with tinnitus, few have investigated how these alterations may change over time. The objective of the current study was to build on the findings in Carpenter-Thompson et al. [5] and Schmidt et al. [10] in order to investigate potential neural plasticity that may be observed when comparing those recently diagnosed with tinnitus (RTIN) to those who have had tinnitus for a long period of time (LTIN).

Few research studies have investigated neural differences between RTIN and LTIN patients. It is important to investigate these differences in order to identify brain regions that may be involved in the initial response to chronic tinnitus and the response to prolonged chronic tinnitus. According to past research, tinnitus may be associated with increased response in the limbic system, specifically the emotional reaction to tinnitus [11–14]. Carpenter-Thompson et al. [5] substantiated these findings by using fMRI and affective sounds to compare neural response across three groups: normal hearing, hearing loss without tinnitus, and hearing loss with tinnitus. Heightened activation in the limbic system, particularly the insula and posterior cingulate, was observed in those with tinnitus compared to matched hearing loss and normal hearing controls. The observed response pattern may be associated with tinnitus distress [5]. Additionally, increased response in frontal regions in those with tinnitus compared to the normal hearing group was noted, which may be associated with enhanced top-down control of emotional processing [15] and habituation to the tinnitus percept (the tinnitus group reported mild form of tinnitus). However, Carpenter-Thompson et al. [5] were unable to comment on the neural plasticity of tinnitus persistence, because those with tinnitus were compared to nontinnitus controls, and all the subjects had experienced tinnitus for longer than one year. Therefore, in the present work, we expanded on Carpenter-Thompson et al.'s [5] study and compared the aforementioned tinnitus patients, referred to as LTIN in this investigation, to individuals recently diagnosed with tinnitus (RTIN).

In addition to earlier task-based analysis [5], a resting state functional connectivity analysis [10] was performed to assess connectivity differences in the same group of participants as Carpenter-Thompson et al. [5]. Resting state functional connectivity analysis allows for the correlation of spontaneous fluctuations in brain activity across regions that reliably form resting state networks. Several recent studies examining these networks in tinnitus patients via fMRI have noted altered connectivity in these patients compared to control groups [13, 16–19]. These changes echo the relationship between limbic and attention regions in tinnitus. In Schmidt et al. [10], network alterations included increased correlations between the primary auditory cortices and the left parahippocampus in tinnitus patients compared to normal hearing controls, reduced correlations between seeds in the default mode network (medial prefrontal cortex and posterior cingulate cortex) and the precuneus in the tinnitus group compared to both control groups, and increased correlation between seeds in the dorsal attention network (bilateral frontal eye fields) and the right parahippocampus in tinnitus compared to hearing loss controls. These changes could also be related to tinnitus habituation, but this study also could not address potential neural plasticity as a result of tinnitus persistence.

The present study used the same experimental paradigms as Carpenter-Thompson et al. [5], which included fMRI imaging and an affective sound categorization task, and the resting state paradigm of Schmidt et al. [10], to evaluate differences in neural correlates between RTIN and LTIN groups. The purpose of the current cross-sectional study was to expand upon the results from our earlier work [5, 10] in order to investigate the neural plasticity of tinnitus persistence. The study focused on two main hypotheses that relate to the possible persistence of mild tinnitus: (1) those with RTIN have increased response in the limbic system associated with an early emotional response to tinnitus, whereas those with LTIN show heightened response in frontal regions reflecting habituation to the tinnitus percept, and (2) subjects with RTIN have altered resting state functional connectivity including increased correlations between the auditory resting state network and limbic areas and a disrupted default mode network compared to LTIN group because they are less able to maintain a true resting state.

## 2. Methods

### 2.1. Subjects

Individuals with tinnitus were recruited from the Urbana-Champaign region of Illinois using flyers, mass emails, and help from local audiological clinics. All participants agreed to participate in the study and provided written informed consent in accordance with UIUC IRB protocol 10144. Monetary compensation was provided to all subjects.

A total of 25 subjects were included in data analysis: 12 individuals with recent onset of tinnitus (RTIN), defined as less than 1 year, and 13 individuals that had tinnitus for more than 1 year (LTIN). RTIN participants were required to have tinnitus for more than 6 months but less than 1 year to ensure it was chronic in nature and recent. Concerning those in the LTIN group, nine participants were able to provide an exact number of years for which they have experienced tinnitus (16.83 ± 15.1) and four participants were unable to provide an exact number of years for which they experienced tinnitus but indicated that they experienced tinnitus for more than 1 year. The RTIN group consisted of 5 male and 7 female participants aged 48.1 ± 10.3, and there were 9 males and 4 females, age 54.7 ± 7.0, in the LTIN group. Groups did not significantly differ in age (*p* = 0.068). All participants completed the tinnitus handicap index (THI) [20], Beck anxiety inventory (BAI) (0–7: minimal, 8–15: mild, 16–25: moderate, and 26–63: severe), and Beck depression inventory (BDI-II) (0–9: minimal, 10–18: mild, 19–29: moderate, and 30–63: severe) [21–24]. In THI, subjects ranged from slightly bothersome tinnitus to mildly bothersome tinnitus. Note that the RTIN group scored higher on average (*p* = 0.012) compared to the LTIN group and a portion of RTIN subjects scored in the mildly bothersome category, but the average score for both groups fell within the slightly bothersome category (Table 1). In BAI, overall, subjects ranged from minimal to mild anxiety (Table 1), with the RTIN group (BAI: 3.7 ± 3.6; 0–13) scoring slightly higher on average compared to the LTIN group (1.7 ± 1.6; 0–3; *p* = 0.026) [25]. In BDI-II, the RTIN group scored between minimal and mild depression and all scores within the LTIN group were within the minimal depression range (Table 1). Similar to the other measures, the RTIN group scored slightly higher on the BDI-II (4.3 ± 3.9; 0–11) compared to the LTIN group (1.3 ± 1.9; 0–6; *p* = 0.024) (Table 1). With regard to hearing loss profiles, all participants in the LTIN group [5] had normal hearing up to 2 kHz and mild-to-moderately severe sloping hearing loss after that and were matched to their own hearing loss controls in Carpenter-Thompson et al. [5] and Schmidt et al. [10] studies. Because of difficulty in recruiting RTIN participants, we chose not to have any criterion with regard to their hearing profiles. Further, seven of the twelve RTIN subjects were inadvertently not assessed with pure tone audiometry. Three of the five RTIN subjects who were assessed demonstrated normal hearing, while the other two had mild-to-moderate hearing loss above 2 kHz as in the LTIN group. One of the two with mild hearing loss also demonstrated a mild loss at 250 Hz in the right ear. However, all RTIN and LTIN subjects included in the study indicated they heard each sound during the scanning session, despite these sounds being broad band in nature; this was further confirmed by their behavioral responses to the sounds via button presses. Additionally, all subjects included in analysis did not have hyperacusis, as assessed by a simple questionnaire. As discussed in the Introduction, the LTIN group was used in previous studies within the lab comparing tinnitus to nontinnitus controls [5, 10].

### 2.2. Stimuli and Task

Stimuli were chosen from the International Affective Digital Sounds (IADS) database and were rated to be pleasant (P), unpleasant (U), or neutral (N) by young healthy adults [26]. Based on the normative scores (9 point scales for valance and arousal with 1 being unpleasant and 9 pleasant and 1 being not arousing and 9 very arousing), 90 sounds were selected: 30 pleasant sounds (valance: 6.83 ± 0.54; arousal: 6.46 ± 0.56), 30 unpleasant sounds (valance: 2.78 ± 0.58; arousal: 6.9 ± 0.56), and, to serve as a baseline during fMRI data analysis, 30 neutral sounds (valance: 4.81 ± 0.43; arousal: 4.85 ± 0.57) [26]. The 90 sound stimuli were presented to the subject through pneumonic headphones (Resonance Technology, Inc., Northridge, CA) while in the fMRI scanner. Presentation of the sounds was controlled using Presentation 14.7 software (http://www.neurobs.com/) on a Windows XP machine. Subjects were asked to rate each sound as either pleasant, unpleasant, or neutral using button presses during the scanning session. Participants were instructed to respond as soon as they felt confident in their rating. Subsequent rating and reaction time data were collected. During fMRI data analysis subject's individual ratings of the sounds were used rather than the normative scores [26], as in Carpenter-Thompson et al. [5].

### 2.3. Data Acquisition

Data were acquired using a Siemens 3T Allegra head only scanner. As with previous work in our lab [5, 27, 28], cluster echo-planar imaging (EPI) acquisition was used to minimize scanner noise interference with stimuli perception [29, 30]. Data from ninety trials, one for each stimulus, were obtained during the emotion task. The repetition time (TR) of each trial was 9 seconds. The 9 s TR interval consisted of 7 s of silence, during which a 6 s sound stimulus was presented, followed by a 2 s scan.

In addition to the emotion task, resting state functional connectivity data were collected. In this scan, continuous scanning was employed for five minutes, during which participants were instructed to lie still and fixate on a cross presented on a screen. One hundred and forty-six volumes were collected and preprocessed for analysis; the first four of the original 150 volumes were discarded to remove the effects of magnet stabilization.

During the scanning session, two sets of anatomical images and two sets of functional images were collected. A structural low-resolution T2-weighted image (AxT2) and a structural high resolution magnetization-prepared rapid-acquisition with gradient echo (MPRAGE) image volumes were collected. Thirty-two low-resolution transversal slices (AxT2) (TR = 7260 ms, TE = 98 ms) were collected for each volume with a 4.0 mm slice thickness and a 0.9 × 0.9 × 4.0 mm^3^ voxel size (matrix size (per slice), 256 × 256; flip angle, 150°). One hundred and sixty MPRAGE 1.2 mm in thickness sagittal slices were obtained for each volume with a 1.0 × 1.0 × 1.2 mm^3^ voxel size (TR, 2300 ms; TE, 2.83 ms; matrix size (per slice), 256 × 256; flip angle, 9°). Functional images of the emotion task were obtained using the following parameters: TR, 9000 ms with 2000 ms acquisition time; TE, 30 ms; slice thickness, 4 mm; interslice gap, 0.4 mm; 32 axial or transverse slices, distance factor, 10%; voxel size, 3.4 × 3.4 × 4.0 mm^3^; field of view read, 220 mm; matrix size (per slice), 64 × 64; flip angle, 90°. Resting state data were collected using a TR of 2000 ms for a total of 150 image volumes for 5 minutes; all other parameters were the same as those for the emotion task.

### 2.4. Data Analysis

#### 2.4.1. Behavior

Behavioral data obtained through in-scanner responses were analyzed using SPSS version 22 software (Statistical Package for Social Sciences, IBM, http://www-01.ibm.com/software/analytics/spss/). Using ANOVA testing with group (RTIN, LTIN) and condition (P, N, and U) set as independent factors and reaction time and rating set as dependent factors, between- and within-group differences were computed. Statistical significance was set at *p* < 0.05 for all behavioral data.

#### 2.4.2. fMRI Data Analysis

Statistical parametric mapping 8 software (SPM8, Welcome Trust Centre for Neuroimaging, http://www.fil.ion.ucl.ac.uk/spm/software/spm8/) was used to analyze the fMRI data. Preprocessing within SPM8 was used to realign, coregister, normalize, and smooth the images. Preprocessing of the resting state data also included slice time correction.* Realignment*. Images were realigned using a rigid body transformation to control for head motion.* Coregistration*. The AxT2 image was coregistered to the mean functional image generated from realignment. The MPRAGE image was then coregistered to the AxT2 image.* Normalization*. The MPRAGE was normalized to match a standard T1 MNI template. The normalization parameters obtained were then applied to the functional images to normalize them into standard MNI space.* Smoothing*. A Gaussian kernel of 8 × 8 × 8 mm^3^ full width at half-maximum was applied.

*Emotion Task*. The smoothed images generated from preprocessing were used in first level fixed effects analysis to generate P > N and U > N contrast images for each subject. Note that the N condition was used as baseline. The contrast images generated from each subject (P > N, U > N) were used in flexible factorial analysis and post hoc independent sample *t*-tests at the second level. The RTIN and LTIN groups were compared directly using whole-brain post hoc independent sample *t*-tests. Supplementary targeted region-of-interest (ROI) analysis using the Wake Forest University Pick atlas toolbox (http://www.fmri.wfubmc.edu) within SPM8 was conducted to select for differences within auditory and limbic regions. An anatomically defined mask of the amygdala, insula, parahippocampus, and auditory cortex (Brodmann areas 42, 41, and 22) was applied to the post hoc independent sample *t*-tests. Auditory regions were included in the analysis since tinnitus has been shown to involve the auditory pathway [9, 31–34]. Statistical significance was set at *p* < 0.025 FWE at the voxel level and small volume correction (SVC) was used for ROI analysis.

*Resting State*. Analysis of the resting state data was performed using the Functional Connectivity Toolbox (Conn) [35] for MATLAB. Following preprocessing, the data were bandpass filtered within Conn from 0.008 to 0.08 kHz. The time series associated with nuisance parameters including white matter, cerebrospinal fluid, and realignment parameters created during preprocessing were regressed out of the data. Next, seed-to-voxel analysis was performed to examine three resting state networks: the auditory resting state network, the default mode network (DMN), and the dorsal attention network (DAN). These networks were chosen based on prior results from our lab that demonstrated connectivity alterations in tinnitus patients when compared to control groups [10]. Multiple seed regions were used to assess the connectivity of each network. In the auditory network, the two seeds were placed in the bilateral primary auditory cortices. In the DMN, seeds were located in the posterior cingulate cortex and the medial prefrontal cortex. The DAN was examined using four seeds located in the bilateral posterior intraparietal sulci and the left and right frontal eye fields (based on [13]). Each seed was a sphere of radius 5 mm created in Marsbar [36]. Coordinates were the same as those used in previous studies in our lab [10, 27] and are listed in Table 2.

For each subject, correlation maps of the whole brain were created for each seed. Then, for each network, the maps were averaged over all seeds to create a single representation of the network. Thus, in the case of the DMN and auditory networks, the single subject correlation map was an average of two seeds, and for the DAN, it was an average of four seeds. These averaged correlation maps were *z*-transformed before group averages were computed to enable cross-group comparisons. Independent sample *t*-tests were performed in Conn [35] and exported to SPM8 for display. Whole-brain analysis was performed; to be significant, clusters needed to be within a *p* < 0.025 FWE corrected threshold at either the cluster or voxel level, with a cluster extent of 20 voxels.

## 3. Results

### 3.1. Behavioral Results

**Figure 1:**
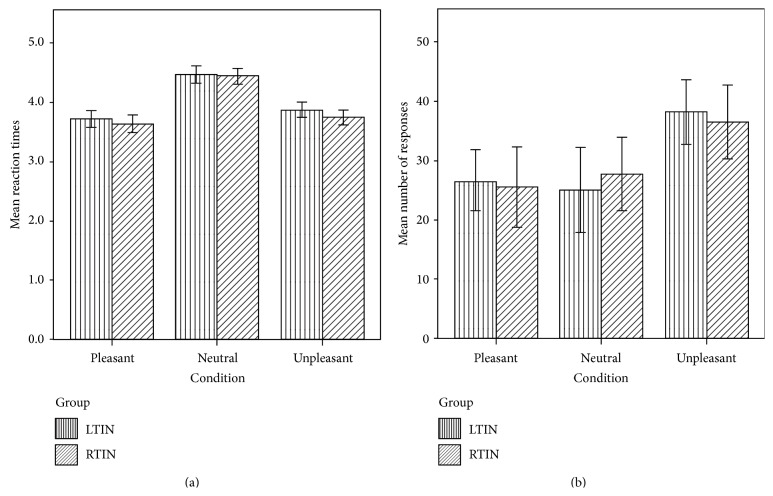
(a) Reaction time results for the emotion task. Both LTIN and RTIN groups responded slower to N sounds compared to P and U sounds. However, there were no significant differences observed between groups. (b) Behavioral response results for the emotion task. Both LTIN and RTIN groups responded more to U sounds than to P sounds. The LTIN group also responded to U sounds more than to N sounds. There were no significant differences observed between groups. Statistical significance *p* < 0.05.

Behavioral data of the emotional task were analyzed using ANOVA tests, with significance set at *p* < 0.05. Group (LTIN, RTIN) and condition (P, N, and U) were used as independent variables and reaction time and responses were used as dependent variables within a general linear model. Both groups responded slower to neutral sounds relative to either pleasant or unpleasant sounds, and upon direct comparison, no statistically significant differences in reaction times between groups were detected (Figure 1(a)). Additionally, both groups responded “unpleasant” significantly more than “pleasant”. The LTIN group responded “unpleasant” significantly more compared to “neutral” (Figure 1(b)). However, when directly comparing groups, no significant differences in sound ratings were observed (Figure 1(b)).

### 3.2. fMRI Results

#### 3.2.1. Emotional Task

A flexible factorial model was used to analyze the main effect of group and main effect of condition. Neural response was observed in the right superior temporal gyrus, left middle frontal gyrus, and left insula for the main effect of group (Table 3). For the main effect of condition, response was observed in the medial frontal gyrus (Table 3). Within-group whole-brain analysis was conducted to confirm that the expected auditory regions were recruited by both groups during the task (Table 4). To investigate between-group differences, we used whole-brain independent sample *t*-tests (Table 5). To better elucidate differences in auditory and limbic regions, subsequent targeted ROI analysis was conducted (Table 6). Note that statistical significance was set to *p* < 0.025 FWE corrected threshold for imaging results.

Consistent with our previous studies, whole-brain analysis revealed heightened response in temporal regions to the affective sounds in both groups [5]. For the LTIN (P > N) contrast increased response was observed in the bilateral superior temporal gyrus, left middle temporal gyrus, left transverse temporal gyrus, and left precuneus (Table 4). Elevated response was observed in the bilateral superior temporal gyrus, right middle temporal gyrus, and left transverse temporal gyrus for the LTIN (U > N) comparison (Table 4). Increased response was observed in the bilateral middle temporal gyrus, left precuneus, and medial frontal gyrus for the RTIN (P > N) contrast (Table 4). For the RTIN (U > N) contrast heightened response was observed in the right middle temporal gyrus, left superior temporal gyrus, and left lentiform nucleus (Table 4).

**Figure 2:**
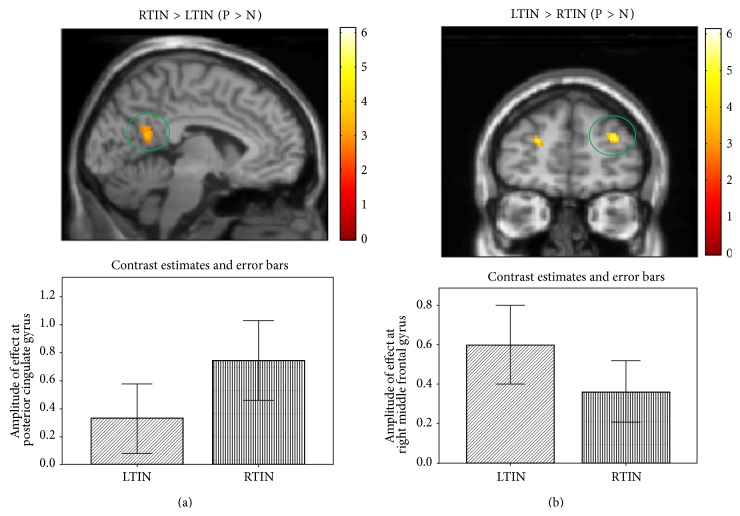
Statistical parametric maps for post hoc independent sample *t*-tests for the emotion task. Whole-brain post hoc independent sample *t-*tests were used to determine group differences. (a) Statistical tests detected increased response in the posterior cingulate in the RTIN group compared to the LTIN group. (b) Statistical tests detected increased response in the right middle frontal gyrus in the LTIN group compared to the RTIN group. For better visualization, the maps are displayed at *p* < 0.001 uncorrected, but the circled cluster is corrected for multiple comparisons (*p* < 0.025 FWE).

Similar to previous studies in our lab, the P > N contrast revealed the greatest number of differences between groups [5]. Using whole-brain independent sample *t*-tests analysis, increased response was observed in the posterior cingulate for the RTIN > LTIN (P > N) contrast (Table 5, Figure 2(a)). Heightened response was observed in the left insula and right precentral gyrus for the RTIN > LTIN (U > N) comparison. To better detect limbic system activation, ROI analysis was implemented and increased left insular response was observed for the RTIN > LTIN (U > N) comparison (Table 6). No suprathreshold voxels were observed for the LTIN > RTIN (U > N) and RTIN > LTIN (P > N) comparisons (Table 6). Increased response was observed in the bilateral middle frontal gyrus (Figure 2(b)), bilateral superior temporal gyrus, right supramarginal gyrus, and cingulate gyrus for the LTIN > RTIN (P > N) comparison. There were no suprathreshold voxels detected for the LTIN > RTIN (U > N) comparison. Upon direct comparison using ROI analysis, increased response was observed in the right superior temporal gyrus for the LTIN > RTIN (P > N) contrast.

#### 3.2.2. Resting State

**Figure 3:**
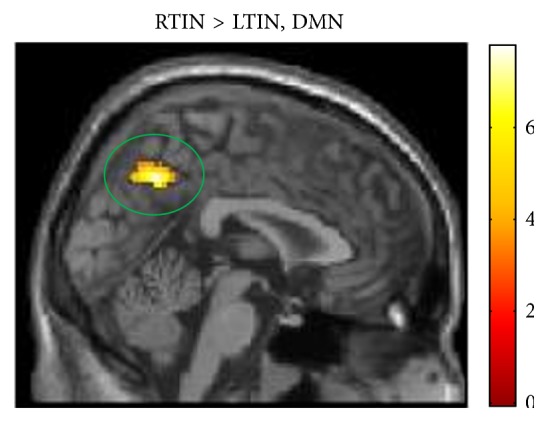
Statistical parametric maps for post hoc independent sample *t*-tests for the resting state analysis of the default mode network. Whole-brain post hoc independent sample *t-*tests were used to determine group differences. Statistical tests detected increased response in the right precuneus/posterior cingulate in the RTIN group compared to the LTIN group. For better visualization, the maps are displayed at *p* < 0.001 uncorrected, but the circled cluster is corrected for multiple comparisons (*p* < 0.025 FWE). DMN: default mode network.

Concerning resting state analysis, there was little difference in functional connectivity between the RTIN and LTIN groups. In the DAN and the auditory network, no regions of significance were located. In the DMN, there was significantly increased connectivity between seeds in the posterior cingulate cortex and medial prefrontal cortex and the right precuneus/posterior cingulate in the RTIN group compared to the LTIN group (Table 7, Figure 3).

## 4. Discussion

Our study produced three main findings concerning fMRI activation and correlation patterns. First, increased activation was observed in the posterior cingulate and insula in those with RTIN compared to LTIN for the unpleasant relative to neutral sounds. Increased posterior cingulate and insula activity may be associated with an initial emotional response to tinnitus. Second, elevated response was found in the middle frontal gyrus in the LTIN group compared to the RTIN group. Increased activation of the frontal regions may indicate habituation to the tinnitus percept over time. Lastly, there was greater resting state functional connectivity between default mode seeds, located in the posterior cingulate cortex (PCC) and the medial prefrontal cortex, and the precuneus/posterior cingulate gyrus in RTIN compared to LTIN patients. This suggests that alterations in the default mode network occur following habituation to the tinnitus stimulus. Together, these findings may indicate an early and a late neural plasticity to chronic internal noise and are discussed in turn.

Consistent with our findings, previous studies have associated abnormal activity in limbic regions, specifically the posterior cingulate and insula, with an emotional reaction to tinnitus resulting in distress [5, 11, 14, 37–39]. Carpenter-Thompson et al. [5] found increased response in the posterior cingulate and insula when comparing those with tinnitus, the current LTIN group, to nontinnitus controls [5]. In the current study, increased response from the posterior cingulate and insula was observed in the RTIN group compared to the LTIN group. Our results suggest that, in early tinnitus, the response from the posterior cingulate and insula is even greater than that of those who have had tinnitus for a long period of time. We suspect that heightened PCC and insular activity in the RTIN group compared to the LTIN group may be neural indicators of an initial emotional reaction to tinnitus. This is corroborated by previous models of tinnitus severity which included insula and cingulate regions as centers of tinnitus distress [5, 40]. Therefore our results support our initial hypothesis that the early reaction to tinnitus may involve heightened activation in limbic regions, specifically the posterior cingulate and insula.

In addition to the limbic activity, increased activation in frontal regions was observed in the LTIN compared to the RTIN group. Consistent with our findings, past studies have found increased activation of frontal regions, including the middle frontal gyrus, in tinnitus [5, 9, 12, 13]. We found increased response in the middle frontal gyrus in the LTIN group compared to the RTIN group. Heightened frontal response has been associated with enhanced top-down control over emotional processing centers. Jacques et al. [15] presented adults with unpleasant images while in an fMRI scanner to measure neural response while viewing the images. They found that increased response from the amygdala was associated with better recall of the unpleasant images, and increased response from frontal regions was associated with poorer recall. The researchers proposed that frontal regions may be used to control emotional response to affective stimuli, thereby ignoring the unpleasant images resulting in poorer recall of the pictures. In our study, increased frontal response may help individuals with tinnitus control their emotional response to affective auditory stimuli. The results also suggest a complex interaction between frontal and limbic regions when processing auditory information that warrants future investigation by other studies. Specifically, elevated response in the middle frontal gyrus may indicate top-down control over the emotional response to the tinnitus percept, resulting in minor improvements in THI scores in LTIN patients relative to RTIN patients.

Evidence for plasticity in the default mode network with tinnitus habituation was also noted in this study. Resting state functional connectivity revealed that an increased correlation between the DMN and the right precuneus was noted in RTIN patients compared to LTIN (Figure 3). This significant cluster is large and encompasses a portion of the posterior cingulate. Thus, there is abnormal limbic activity at rest in RTIN patients that is not present in LTIN patients. It is possible that this alteration, as suggested in the task-based results, reflects a heightened emotional response to the percept because the RTIN group has not yet habituated to it. However, reduced correlation between the same DMN and the precuneus/posterior cingulate was observed in Schmidt et al. [10], where LTIN patients were compared to both normal hearing and hearing loss controls. Therefore, it is likely that alterations to resting state networks occur later than task-related alterations in tinnitus patients, and the results seen in this study reflect the same decreased coherence in the LTIN previously noted [10], as opposed to abnormal increases in correlations in the RTIN group. Thus, the reduced coherence of the DMN in LTIN could be indicative of neural plasticity as a consequence of tinnitus habituation. Those with recent onset of tinnitus may have not yet had time to establish these altered functional connectivity patterns at rest.

## 5. Caveats

The present study was a cross-sectional study, rather than a longitudinal study, and therefore we can only speculate as to the meaning of neural differences between LTIN and RTIN groups. We suggest a longitudinal functional imaging study of tinnitus in order to better identify the functional changes in tinnitus over time and to make more substantial claims as to which brain areas are involved in the plasticity of tinnitus persistence. Additionally, the present study did not account for possible differences in hearing loss in the two groups. Past studies in our lab have suggested that hearing loss may contribute to the observed functional results [5, 27]. Furthermore, it is unknown whether the RTIN participants will develop more severe forms of tinnitus or habituate to the percept which would be elucidated in a longitudinal study. Nevertheless, this study serves as a baseline for future studies to build upon to better understand how tinnitus may alter brain response patterns and functional connectivity over time. Additionally, the inclusion of more recent questionnaires such as the Tinnitus Functional Index [41] or Tinnitus Primary Functional Questionnaire [42] may allow for more robust measurements of distress such as subscores including sleep impairments and depression levels. Another caveat is that some types of long-term neural plasticity result in severe forms of tinnitus. Here we have only considered mild tinnitus of long duration; it is likely that persistent severe tinnitus may result in different patterns of activation and functional connectivity.

## 6. Conclusion

We found evidence for differences in neural correlates between RTIN and LTIN individuals suggesting that the limbic system may play a role in an initial emotional response to tinnitus, whereas frontal regions and default mode network alterations may be involved in habituation to tinnitus. In support of this model, we found increased activation in the posterior cingulate and insula in the RTIN group compared to the LTIN group during the emotion task. Further supporting this model, the LTIN group showed heightened response in the frontal regions compared to RTIN participants and increased correlations between the default mode network and the precuneus in RTIN compared to LTIN, suggesting that decreased coherence of the network is associated with tinnitus habituation. Increased response in the frontal regions may be employed by those with persistent chronic tinnitus to better control their emotional response and facilitate habituation to tinnitus.

## Figures and Tables

**Table 1:** Demographic and clinical characteristics for both groups. THI: tinnitus handicap inventory; BAI: Beck anxiety inventory; BDI-II: Beck depression inventory. Age, THI, BAI, and BDI-II scores were compared using independent sample *t*-tests. *∗* indicates significant difference between groups at *p* < 0.05.

Group	RTIN (recent tinnitus)	LTIN (long-term tinnitus)
Group size	12	13
Age (M ± SD)	48.1 ± 10.3; 32–65	54.7 ± 7.0; 42–64
Gender	5 male, 7 female	9 male, 4 female
Handedness	11 right, 1 left	12 right, 1 left
BAI (M ± SD)	3.7 ± 3.6; 0–13^*∗*^	1.7 ± 1.6; 0–3^*∗*^
BDI-II (M ± SD)	4.3 ± 3.9; 0–11^*∗*^	1.3 ± 1.9; 0–6^*∗*^
THI (M ± SD)	15.7 ± 10.2; 2–34^*∗*^	8.3 ± 6.8; 0–18^*∗*^

**Table 2:** List of seed regions used during the resting state connectivity analysis. DMN: default mode network; DAN: dorsal attention network.

Network	Seed region	MNI coordinates *X*, *Y*, *Z*
Auditory	Right primary auditory cortex	55, −22, 9
Auditory	Left primary auditory cortex	−41, −27, 6
DMN	Medial prefrontal cortex	8, 59, 19
DMN	Posterior cingulate cortex	−2, −50, 25
DAN	Left posterior intraparietal sulcus	−23, −70, 46
DAN	Right posterior intraparietal sulcus	26, −62, 53
DAN	Left frontal eye field	−25, −11, 54
DAN	Right frontal eye field	27, −11, 54

**Table 3:** Local maxima for the main effect of group and condition in the emotion task experiment. Regions are listed in Montreal Neurological Institute (MNI) coordinates. Brodmann areas are also provided (before determining the Brodmann areas, the MNI coordinates were converted to Talairach coordinates). Statistical threshold was set at *p* < 0.025 FWE corrected for multiple comparisons. L: left; R: right.

Contrast	MNI coordinates *X*, *Y*, *Z*	*Z* score	Cluster mm^3^	Brain region (Brodmann area)
Main effect group	56, −40, 12	5.19	648	R. superior temporal gyrus (BA 22)
	−40, 2, 40	5.14	234	L. middle frontal gyrus (BA 9)
	−34, −10, 16	5.02	330	L. insula (BA 13)

Main effect condition	0, 52, −4	5.31	1444	Medial frontal gyrus (BA 10)

**Table 4:** Local maxima for the whole-brain analysis for within-group contrasts in the emotion task experiment. Whole-brain analysis for both P > N and U > N conditions was computed for each group. Regions are listed in Montreal Neurological Institute (MNI) coordinates. Brodmann areas are also provided (before determining the Brodmann areas, the MNI coordinates were converted to Talairach coordinates). Statistical threshold was set at *p* < 0.025 FWE corrected for multiple comparisons. L: left; R: right; P: pleasant; N: neutral; U: unpleasant.

Contrast	MNI coordinates *X*, *Y*, *Z*	*Z* score	Cluster mm^3^	Gyrus (Brodmann area)
LTIN group (P > N)	54, −16, 2 52, −34, 4 66, −30, 0	6.14 6.06 5.46	273	R. superior temporal gyrus (BA 22) R. middle temporal gyrus (BA 22) R. middle temporal gyrus (BA 21)
	−54, −10, 0 −56, −16, 10 −56, −2, −2	5.94 5.68 5.52	303	L. superior temporal gyrus (BA 22) L. transverse temporal gyrus (BA 42) L. superior temporal gyrus (BA 22)
	−62, −40, 10	5.35	43	L. superior temporal gyrus (BA 22)
	−6, −56, 34 −10, −48, 30	5.28 5.02	61	L. precuneus (BA 7) L. precuneus (BA 31)

LTIN group (U > N)	−58, −8, 2 −58, −16, 10	5.81 5.61	371	L. superior temporal gyrus (BA 22) L. transverse temporal gyrus (BA 42)
	54, −46, 2 58, −4, 0 60, 2, −6	5.69 5.34 5.26	194	R. middle temporal gyrus (BA 22) R. superior temporal gyrus (BA 22) R. superior temporal gyrus (BA 22)
	48, −34, 4	5.34	29	R. superior temporal gyrus (BA 22)

RTIN group (P > N)	58, 2, −8 56, −10, −4	6.12 5.19	103	R. middle temporal gyrus (BA 21) R. middle temporal gyrus (BA 22)
	−10, −46, 44	5.74	22	L. precuneus (BA 7)
	−54, −20, −6	5.67	35	L. middle temporal gyrus (BA 21)
	14, 50, 10	5.32	30	Medial frontal gyrus (BA 10)

RTIN group (U > N)	−26, −6, −6	5.95	45	L. lentiform nucleus
	58, 2, −8	5.78	46	R. middle temporal gyrus (BA 21)
	−54, −18, −6	5.62	25	R. middle temporal gyrus (BA 21)
	−54, −6, 4 −56, 0, −4	5.11 5.05	44	L. superior temporal gyrus (BA 22) L. superior temporal gyrus (BA 22)

**Table 5:** Local maxima for the whole-brain independent sample *t*-tests in the emotion task experiment. Whole-brain independent sample *t*-tests were computed for between-group differences. Regions are listed in Montreal Neurological Institute (MNI) coordinates. Brodmann areas are also provided (before determining the Brodmann areas, the MNI coordinates were converted to Talairach coordinates). Statistical threshold was set at *p* < 0.025 FWE corrected for multiple comparisons (*∗* indicates significance at cluster level only). L: left; R: right.

Contrast	MNI coordinates *X*, *Y*, *Z*	*Z* score	Cluster mm^3^	Gyrus (Brodmann area)
LTIN > RTIN (P > N)	−16, −12, 32	5.13	117	L. middle cingulate gyrus
	52, −42, 16	5.10	596	R. superior temporal gyrus (BA 22)
	54, 18, 30	5.00	348	R. middle frontal gyrus (BA 46)
	42, −50, 26	4.85	154^*∗*^	R. supramarginal gyrus (BA 40)
	−40, 2, 40	4.83	246^*∗*^	L. middle frontal gyrus (BA 9)
	6, 12, 30	4.82	145^*∗*^	R. middle cingulate gyrus
	−64, −38, 10	4.81	603^*∗*^	L. superior temporal gyrus (BA 22)
	−22, −12, 52	4.58	248^*∗*^	L. middle frontal gyrus (BA 6)

LTIN > RTIN (U > N)				No suprathreshold voxels

RTIN > LTIN (P > N)	12, −66, 10	4.58	215^*∗*^	Posterior cingulate gyrus

RTIN > LTIN (U > N)	−32, −20, 12	4.82	365^*∗*^	L. insula
	38, −12, 36	4.81	265^*∗*^	R. precentral gyrus (BA 4)

**Table 6:** Local maxima for the region-of-interest (ROI) independent sample *t*-tests in the emotion task experiment. Between-group independent sample *t*-tests using ROI analysis comprised of amygdala, insula, parahippocampus, and auditory cortex (Brodmann areas 42, 41, and 22) were conducted. Regions are listed in Montreal Neurological Institute (MNI) coordinates. Brodmann areas are also provided (before determining the Brodmann areas, the MNI coordinates were converted to Talairach coordinates). Statistical threshold was set at *p* < 0.025 FWE corrected for multiple comparisons (*∗* indicates significance at cluster level only). L: left; R: right.

Contrast	MNI coordinates *X*, *Y*, *Z*	*Z* score	Cluster mm^3^	Gyrus (Brodmann area)
LTIN > RTIN (P > N)	50, −42, 18	4.66	34	R. superior temporal gyrus (BA 22)
LTIN > RTIN (U > N)				No suprathreshold voxels
RTIN > LTIN (P > N)				No suprathreshold voxels
RTIN > LTIN (U > N)	−32, −20, 14	4.46	216^*∗*^	L. insula (BA 13)

**Table 7:** Regions of significance in the resting state connectivity analysis. Between-group independent sample *t*-tests were performed for each resting state network examined. Regions are listed in Montreal Neurological Institute (MNI) coordinates. Brodmann areas are also provided where applicable. Statistical threshold was set at *p* < 0.025 FWE corrected for multiple comparisons. Aud: auditory resting state network; DAN: dorsal attention network; DMN: default mode network; R: right.

Contrast	MNI coordinates *X*, *Y*, *Z*	*Z* score	Cluster mm^3^	Gyrus (Brodmann area)
Aud RTIN > LTIN	No suprathreshold voxels			
Aud LTIN > RTIN	No suprathreshold voxels			
DAN RTIN > LTIN	No suprathreshold voxels			
DAN LTIN > RTIN	No suprathreshold voxels			
DMN RTIN > LTIN	2, −58, 42	5.38	199	R. precuneus/posterior cingulate
DMN LTIN > RTIN	No suprathreshold voxels			

## Abbreviations

THI: Tinnitus handicap inventory
BAI: Beck anxiety inventory
BDI-II: Beck depression inventory
LTIN: Patients with tinnitus for longer than 1 year
RTIN: Patients diagnosed with tinnitus within 1 year
P: Pleasant
N: Neutral
U: Unpleasant
fMRI: Functional magnetic resonance imaging
EEG: Electroencephalography
TR: Repetition time.
